# Complete Genome Sequence of *Kosakonia sacchari* Strain BO-1, an Endophytic Diazotroph Isolated from a Sweet Potato

**DOI:** 10.1128/genomeA.00868-16

**Published:** 2016-09-08

**Authors:** Rina Shinjo, Kazuma Uesaka, Kunio Ihara, Kseniia Loshakova, Yuri Mizuno, Katsuya Yano, Aiko Tanaka

**Affiliations:** a Graduate School of Bioagricultural Sciences, Nagoya University, Chikusa, Nagoya, Japan; b Center for Gene Research, Nagoya University, Chikusa, Nagoya, Japan

## Abstract

The complete genome sequence of the endophytic diazotroph *Kosakonia sacchari*, isolated from a sweet potato, was analyzed. The 4,902,106-bp genome with 53.7% G+C content comprises 4,638 open reading frames, including *nif* genes, 84 tRNAs, and seven complete rRNAs in a circular chromosome.

## GENOME ANNOUNCEMENT

The endophytic bacterial strain BO-1 was originally isolated from the surface-sterilized stem of a sweet potato (*Ipomoea batatas*, cultivar “Beniotome”) from Miyakonojo, Miyazaki, Japan, and identified as *Klebsiella oxytoca* based on a partial sequence of the 16s rRNA (1). The strain grew diazotrophically in a free-living state under nitrogen-deficient conditions and showed a positive reaction in the acetylene reduction activity test (1). Whole-genome sequencing analysis revealed that the genome of strain BO-1 is most similar to *Kosakonia sacchari* strain SP1, isolated from sugarcane (2); therefore, this strain likely belongs to *Kosakonia sacchari*, which is a Gram-negative, aerobic, non-spore-forming, motile rod-shaped species, recently reclassified from *Enterobacter sacchari* (3).

The bacterial strain BO-1 (MAFF 211344) was obtained from the National Institute of Agrobiological Science (NIAS, Japan). Genomic DNA was extracted using a genomic DNA purification kit (Promega, USA). Paired-end DNA libraries were prepared for sequencing with an Illumina MiSeq platform. A total of 12,874,059 high-quality paired-end reads were generated, with 788-fold coverage. *De novo* assembly was performed using SPAdes genome assembler version 3.6.2 (4). Removal of short contigs resulted in 916 contigs with an *N*_50_ of 828,908 bp. Gaps were closed by PCR amplification and Sanger sequencing of the PCR products. The genome of *K. sacchari* BO-1 consists of a circular chromosome (4,902,106 bp; 53.7% G+C content).

The strain BO-1 is most closely related to *K. sacchari* strain SP1, with which it shares 98.67% average nucleotide identity (ANI) (5). Since two genomes with an ANI >95% are considered to be from the same species, the strain BO-1 is reidentified as *Kosakonia sacchari*. The chromosome of *K. sacchari* strain SP1 had the highest sequence similarities to the chromosome of *Enterobacter* sp. strain R4-368, an endophytic nitrogen-fixing bacterium isolated from *Jatropha curcas* (2, 6). The ANIs of *Enterobacter* sp. strain R4-368 with *K. sacchari* strain BO-1 and strain SP1 were 94.06% and 94.01%, respectively.

The genomic sequences were annotated using the NCBI Prokaryotic Genome Annotation Pipeline (http://www.ncbi.nlm.nih.gov/genome/annotation_prok). The chromosome has 4,448 coding sequences, seven sets of rRNA genes, and 84 tRNA genes. The genome annotation also confirmed the presence of the *nif* gene locus for nitrogen fixation.

### Accession number(s).

The genome sequence of *K. sacchari* strain BO-1 was deposited in DDBJ/EMBL/GenBank under the accession number CP016337.
